# The Expressed MicroRNA—mRNA Interactions of *Toxoplasma gondii*

**DOI:** 10.3389/fmicb.2017.02630

**Published:** 2018-01-04

**Authors:** İlhan E. Acar, Müşerref D. Saçar Demirci, Uwe Groß, Jens Allmer

**Affiliations:** ^1^Biotechnology, Izmir Institute of Technology, Izmir, Turkey; ^2^Molecular Biology and Genetics, Izmir Institute of Technology, Izmir, Turkey; ^3^Medical Microbiology, Universitätsmedizin Göttingen, Göttingen, Germany

**Keywords:** *Toxoplasma gondii*, microRNA, regulation, miRNA target, miRNA-mRNA interactions, expression analysis, differential expression

## Abstract

MicroRNAs (miRNAs) are involved in post-transcriptional modulation of gene expression and thereby have a large influence on the resulting phenotype. We have previously shown that miRNAs may be involved in the communication between *Toxoplasma gondii* and its hosts and further confirmed a number of proposed specific miRNAs. Yet, little is known about the internal regulation via miRNAs in *T. gondii*. Therefore, we predicted pre-miRNAs directly from the type II ME49 genome and filtered them. For the confident hairpins, we predicted the location of the mature miRNAs and established their target genes. To add further confidence, we evaluated whether the hairpins and their targets were co-expressed. Such co-expressed miRNA and target pairs define a functional interaction. We extracted all such functional interactions and analyzed their differential expression among strains of all three clonal lineages (RH, PLK, and CTG) and between the two stages present in the intermediate host (tachyzoites and bradyzoites). Overall, we found ~65,000 expressed interactions of which ~5,500 are differentially expressed among strains but none are significantly differentially expressed between developmental stages. Since miRNAs and target decoys can be used as therapeutics we believe that the list of interactions we provide will lead to novel approaches in the treatment of toxoplasmosis.

## Introduction

It is estimated that more than 30% of the world population are chronically infected with the protozoan parasite *Toxoplasma gondii*. Upon infection with either oocysts or tissue cysts containing bradyzoites, the parasite converts into the replicative tachyzoite stage that may cause harm especially in fetuses (Jones et al., 2003; de Moraes et al., 2011) and immunocompromised patients (Contini, 2008). In contrast, infection is mostly asymptomatic in immunocompetent individuals, where reconversion into the bradyzoite stage results in the formation of persistent tissue cysts. These are mainly located in neurons and skeletal muscle cells (Schlüter et al., 2014). The interconversion between the bradyzoite and tachyzoite stages is based on a complex interaction between the parasite and its host cell and involves, besides others, immunologic, epigenetic, and cell cycle factors (Sullivan and Jeffers, 2012; Swierzy and Lüder, 2015). Most recently, two stress-induced ApiAP2 transcription factors have been identified which have opposite functions in bradyzoite development (Hong et al., 2017). So far, existing treatment has major limitations and targets only the replicative tachyzoite stage by mainly inducing tachyzoite-bradyzoite conversion thereby promoting parasite persistence (Montazeri et al., 2017). MicroRNAs (miRNAs) have been established as disease targets (Avci and Baran, 2014) and miRNA mimics as drugs (Wang, 2011) and they may be of use to target toxoplasmosis. MicroRNAs are short non-coding sequences involved in post-transcriptional regulation of protein expression. We and others previously analyzed the possible miRNAs of *T. gondii* (Cakir and Allmer, 2010; Wang et al., 2012) and how they could be useful to modulate host protein abundance (Saçar Demirci et al., 2014, 2016). Here we employ the state of the art in pre-miRNA detection from the *T. gondii* genome and also establish putative mRNA targets as well as miRNA and mRNA expression. Thereby, we establish expressed interactions (~65,000) from overall ~2,500 expressed miRNAs and ~8,500 expressed mRNAs. MicroRNAs are only functional when co-expressed with their targets and, therefore, it is important to focus on expressed interactions and this is the first time these have been established in *T. gondii*. Such interactions convey function and thus can provide leads for disease markers and drugs to tackle this global disease.

## Materials and methods

### Data

The majority of human clinical cases of toxoplasmosis in Europe and North America are caused by type II strains (Sibley et al., 2009). The reference genome for *T. gondii* type II strains (ToxoDB-25_TgondiiME49) was downloaded from toxodb.org (Gajria et al., 2007) along with its annotation for known genes and transcripts.

*T. gondii* RNA-Seq data (Croken et al., 2014) was downloaded from the sequence read archive (Leinonen et al., 2011). Accession numbers for the downloaded data ranged from SRR1542919 to SRR1542936 (Table 1). The study contained samples from three different *T. gondii* strains representing the three most abundant clonal types I, II, and III (RH, PLK, and CTG). For each strain tachyzoites and bradyzoites were measured in triplicates induced in cell culture by pH 7 and 8 (Croken et al., 2014), respectively. Previously described mature miRNAs (Wang et al., 2012) were downloaded from their supplementary data and were used to obtain their corresponding hairpins sequences and to train suitable pre-miRNA detection models.

**Table 1:** Sequencing reads statistics for all 18 samples downloaded from SRA.

**Samples**	**Raw reads**	**Cleaned reads**	**Max. clean read length**	**Deleted reads (%)**	**Reads mapped on human (%)**	**Toxo mapped reads (%)**
SRR1542919	58,730,137	2,077,159	50	96.46	1.05	84.49
SRR1542920	43,055,026	11,581,307	50	73.10	0.91	72.09
SRR1542921	44,958,415	14,617,199	75	67.49	0.70	75.73
SRR1542922	43,264,535	1,375,655	50	96.82	0.75	85.54
SRR1542923	53,074,533	13,476,636	50	74.61	1.01	69.58
SRR1542924	55,077,053	18,329,562	75	66.72	0.77	76.15
SRR1542925	36,994,224	1,423,290	50	96.15	3.27	88.33
SRR1542926	48,595,529	12,544,189	50	74.19	1.25	77.00
SRR1542927	55,934,799	17,482,709	75	68.74	1.12	81.83
SRR1542928	74,716,539	2,727,669	50	96.35	3.20	89.18
SRR1542929	51,517,301	12,437,480	50	75.86	2.61	79.16
SRR1542930	41,089,401	13,451,657	75	67.26	1.42	83.09
SRR1542931	211,425,021	7,886,857	50	96.27	1.89	87.22
SRR1542932	44,043,513	9,678,918	50	78.02	2.06	68.16
SRR1542933	248,076,128	80,172,404	75	67.68	1.88	78.25
SRR1542934	51,790,061	1,704,048	50	96.71	2.26	83.78
SRR1542935	55,535,624	14,076,802	50	74.65	4.72	71.03
SRR1542936	38,718,995	13,031,003	75	66.34	3.74	75.64
Mean	69,810,935	13,781,919	58	80	2	79

*The number of reads before and after pre-processing, final average read length as well as mapping ratios to human and T. gondii present important information to assess the quality of the measurement. Additional details can be found in Supplementary Table 1*.

### Pre-miRNA detection

The hairpins obtained in a previous work by fragmenting the whole genome of *T. gondii* (Saçar Demirci et al., 2016), were used for this analysis. Features of hairpins were calculated on AWS (Amazon Web Services) using an in-house java package; but could also be calculated using online services (Yones et al., 2015; Bağci and Allmer, 2016). For creating models specific to *T. gondii*, the izMiR framework (Allmer and Saçar Demirci, 2016) was used at 1,000-fold Monte Carlo Cross Validation (Xu and Liang, 2001) with 70% data for training and 30% data for testing, sampled at equal amounts. Six hundred and eighty three putative pre-miRNAs from our previous studies (Saçar Demirci et al., 2016) containing the published 339 known mature miRNAs (Wang et al., 2012) were used as positive examples during the training of the classifier while pseudo pre-miRNAs (Ng and Mishra, 2007) were applied as negative ones. For obtaining the positive dataset, pre-miRNAs of *T. gondii* were extracted from its genome by extending known mature miRNA sequences by 50 nucleotides to both directions and thereby extracting pre-miRNAs. The new models and the existing izMiR models based on human miRNA data, were applied to all of the extracted hairpins from the *Toxoplasma* genome ME49.

### Mature miRNA detection

Since only few (339) mature miRNAs have been proposed for *T. gondii* (Wang et al., 2012), a general prediction model was created by using all mature miRNAs listed in miRTarBase (Release 6.0) which corresponded to 4316 mature miRNA sequences in miRBase. A negative data set was created by shifting the mature sequences by half of their length within the hairpin sequences (Gkirtzou et al., 2010). To describe mature miRNAs 101 features were calculated: start and end positions of mature sequence (2), central loop start and end points (2), hairpin length, miRBase hairpin length, stem length, mature length, maximum loop length (5), number of matches and mismatches in the mature sequence region (2), single nucleotide counts (4), dinucleotide counts (16), trinucleotide counts (64), distances of start and end positions to 3′, 5′, loop start and loop end (6). These data sets were used to train a random forest learner using 1,000-fold MCCV and 70% (learning) to 30% (testing) ratio (Supplementary Figure 1). The model with the highest accuracy score (0.932) was applied to predicted miRNA hairpins.

### MicroRNA targeting

MicroRNAs function by providing a complementary sequence to parts of their target mRNA. This can be predicted by diverse computational tools. Here, psRNATarget (2011 release) (Dai and Zhao, 2011) was used to establish miRNA—mRNA interactions using default settings. While psRNATarget originally focused on plant miRNA targeting, it now also includes metazoan mRNAs for targeting as well as the possibility to provide miRNAs and putative targets directly. MicroRNA regulation seems to be metazoan like in *T. gondii* (Braun et al., 2010) and, therefore, psRNATarget seemed suitable for this analysis and the default settings were used for the analysis of *T. gondii*. All of the genes of the available transcript annotations for *T. gondii* were used as target sites since UTRs have not been established.

### Expression analysis

#### Gene expression

The RNA-seq data downloaded from the sequence read archive (SRA) at NCBI concerned the accessions ranging from SRR1542919 to SRR1542936 (Table 1). Reads in the associated measurements were first cleaned by removing adapters, low quality regions, or complete reads using Cutadapt (Martin, 2011) and Sickle (Joshi and Fass, 2011). The quality score cutoff was set to 30 in respect to quality trimming, and the minimum length of read threshold was set to 30. Following cleaning of the reads, they were mapped to the human reference genome (GRCh38) using Tophat (v1.4.1) (Trapnell et al., 2009) to filter out any human contaminant reads. Then, unmapped reads were mapped onto the *T. gondii* reference genome ME49. Because of the colorspace characteristics of the samples, a large amount of reads were filtered and an older version of Tophat had to be used instead of current version v2.1.1. The annotation file for the *T. gondii* genome (ToxoDB-25_TgondiiME49) was downloaded from toxodb.org along with its genome. An in-house script was used to count mapped reads (https://github.com/erkinacar5/SortedNucleotideCounter). Genes with less than five reads mapped onto them were considered as not expressed and filtered accordingly. A new normalization method, nucleotide normalization (see “Normalization” section), was adopted to normalize mapped read counts. Normalized counts were further filtered by their expression among strains and developmental stages. Genes that were not expressed in at least 70% of the samples of their respective strain or developmental stage were filtered out. The R platform (R Core Team, 2016) was used to establish expression differences between strains and developmental stages as well as to perform statistical analysis (see below).

#### MicroRNA expression

A custom annotation file for pre-miRNAs predicted in this study was created from the pre-miRNA locations in the genome. With this annotation file it became possible to employ standard workflows to establish miRNA abundance (see gene expression). Counting was done by the mentioned in-house java code. Normalization was done by nucleotide normalization method (see “Normalization” section). Due to lower amount of mapping to miRNA regions, miRNAs with lower than two reads mapped onto them were considered as not expressed and were discarded. Similar to gene expression, if a miRNA was not expressed in at least 70% of the samples in their respective strains or developmental stages it was filtered out.

#### MicroRNA mRNA interactions

An interaction, in this study, is defined as a miRNA co-expressed with at least one of its target mRNAs in the same sample. MicroRNAs often originate from genes and these source genes can be used to extend the interaction to the gene level by associating them with metabolic or regulatory pathways. Thus a complete interaction is defined by the source gene, miRNA, and its target gene(s). MicroRNAs that did not come from a known gene were filtered out in this study. Biologically, these miRNAs may be of interest, however, it cannot be shown that they lead to expressed interactions which are of prime interest for this study. Future research may shed light on miRNAs that derive from their own loci.

#### Normalization

Generally, RPKM (reads per kilobase per million mapped reads) or FPKM (fragments per kilobase per million mapped reads) are used to allow comparison of transcript expression among measurements. However, the source data showed large differences in average read length. Therefore, it was necessary to take into account the actual amount of mapped nucleotides as well as transcript lengths in terms of nucleotides per kilobase of transcript per million nucleotides mapped (NKMN). An in-house java code (https://github.com/erkinacar5/SortedNucleotideCounter) allowed mapped reads to be counted including their read length. With this information, it was possible to calculate total nucleotides mapped onto specific regions of a genome. Using the annotation files, for all the samples, total nucleotides of each transcript (gene or miRNA) were calculated as well as total amount of nucleotides mapped to each transcript. Normalization was done by total mapped nucleotides per gene or miRNA divided by total nucleotide number of corresponding gene or miRNA and total nucleotides in the sample. Since the ratio was aimed to show per kilobase per million, it was multiplied by a billion to bring the values into an intuitive range:

$$\begin{array}{l} \frac{\Sigma \left(Mapped\;nucleotides\;on\;gene\;or\;miRNA\right)\times \;1.000.000.000}{\;\Sigma \left(Nucleotides\;of\;gene\;or\;miRNA\right)\;\times \;\Sigma \left(Nucleotides\;in\;sample\right)\;} \end{array}$$

For the interactions, an interaction ratio was computed by dividing target gene expression amount by targeting miRNA expression amount. Then, this interaction ratio was normalized by the sample median for interaction ratios. After this, the median value of all medians was taken and each normalized sample was divided by median of median values for final normalization.

#### Differential expression analysis

Differential expression analyses for gene and miRNA expression, as well as for interactions was done in R. Ratios of normalized expression values between strains (RH vs. PLK, RH vs. CTG, and PLK vs. CTG) and between developmental stages (tachyzoite vs. bradyzoite) were converted to log2 fold changes. Student's *t*-test was performed in R for each gene, miRNA or interaction among different strains and developmental stages. *P*-values were derived from these tests and adjusted according to Benjamini and Hochberg (1995). The average read length was different among samples (Table 1) and this affected miRNA expression. Thus, counts and their normalization (Supplementary Figure 2), expressions of their source genes were used to represent their abundance in interactions. Moreover, to assess interactions, the ratio between targeted gene expression and source gene expression was established. Interaction ratios were calculated on a per sample basis if both source and target expressions were larger than zero and had at least five reads.

### Annotation of genes and MicroRNAs

The gene annotations for *T. gondii* were downloaded from toxodb.org and they contained gene names in form of a combination of strain and accession number (e.g., gene TGME49_293600). To make annotations clearer, unigene identifiers were used where possible. In cases where a protein is synthesized from multiple genes, gene accessions (e.g., 293600) were added to the end of the protein name (e.g., RPL27_293600). For genes that did not have known protein products, BLAST (v2.4.0+) (Camacho et al., 2009) was used to align these genes with genes of other *T. gondii* strains. Similarity above 80% (with mismatch and gap <4) were accepted as annotated. Those with no similarity and no known proteins were left unchanged and the toxodb.org accession was used. Predicted miRNAs were annotated with their source strand (Pos for positive and Neg for negative), genomic start position and their respective chromosome, super contig, or contig name (e.g., Neg_263687_TGME49_chrII). Further annotation was done by aligning all miRNAs in miRBase and the 339 *T. gondii* specific previously detected mature miRNAs to our predicted pre-miRNAs using BLAST. For similarity above 75%, names of the aligned pre-miRNAs were cross annotated. Our accession numbers were added to these miRNAs to be able to track their sources. MicroRNAs below 75% similarity were left unchanged. To be able to differentiate among mature miRNAs originating from different genomic regions, a number was attached to their identifier (e.g., Neg_263687_TGME49_chrII_2).

## Results and discussion

### MicroRNA detection

In a previous study (Saçar Demirci et al., 2016) we folded the *T. gondii* ME49 genome and extracted all hairpin like structures (~5 million). Since many of these hairpins are unlikely to be pre-miRNAs, we trained a machine learning model using our izMiR framework (Allmer and Saçar Demirci, 2016) and used it for the assessment of the putative hairpins. In total, 1,227,917 hairpins were analyzed and these were filtered by their confidence scores (>0.99) using the izMiR model. This filtering resulted in 4,589 confident pre-miRNAs. We further required the pre-miRNAs to be part of a gene and did not take into account intergenic pre-miRNAs. About 300 candidate pre-miRNAs were affected by this filtering leaving 4,240 hairpins for further analyses. Expression with at least two mapped reads in at least one of the samples was the final requirement for pre-miRNAs and 2,484 passed this filtering step (Supplementary Table 2). Pre-miRNAs are further processed into mature miRNAs in the miRNA genesis pathway and this process was mimicked by fragmenting the 4,589 confident candidate hairpins into 24 nucleotide (nt) long sequences with 6 nt overlaps. Since the length of mature sequences is generally smaller or equal to 24, the majority of mature sequences should be in the generated candidate pool. This pool is too large for further analysis and, therefore, a machine learning model was established to discriminate among candidates. Mature candidates with a minimum of 15 nt long sequences and a model prediction score of at least 1.0 were used for further analysis. 4,234 mature sequences passed the filtering by the machine learned model (Supplementary Table 3). Among these, 973 mature sequences overlap with the hairpin loop and were removed while 89 include the complete loop and were retained as loop-miRs (Winter et al., 2013). 1,058 mature miRNAs are located on the 5′ arm while 2,114 are located on the 3′ one. The confident pre-miRNA candidates were compiled into a genome feature format file to enable their expression analysis using standard workflows. In the following gene expression and miRNA expression among various *T. gondii* strains and developmental stages will be discussed.

### Gene expression

**Figure 1:**
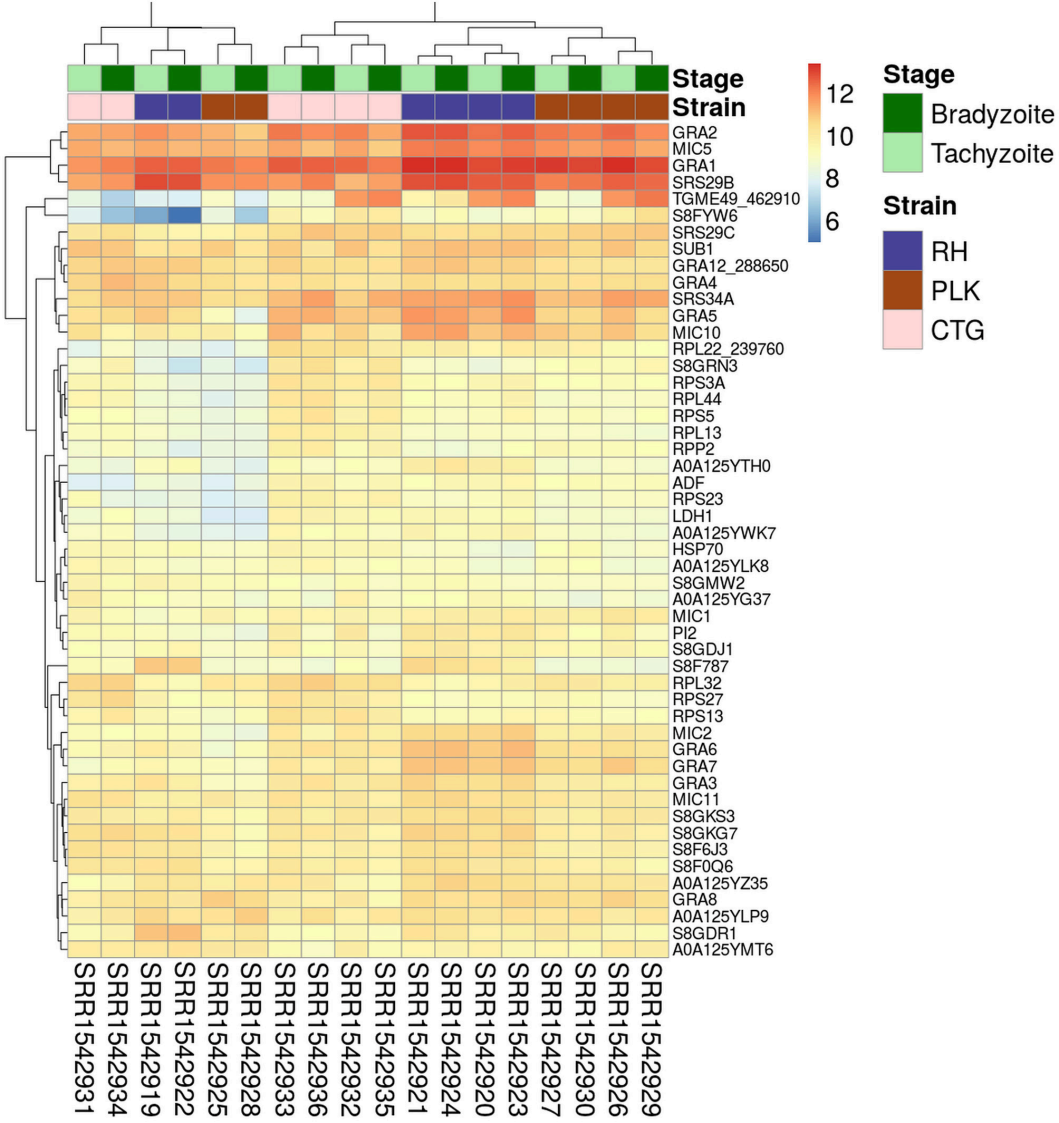
Heatmap showing the 50 genes with largest average expression among samples. The strains (type I RH: blue, type II PLK: brown, and type III CTG: pink) and developmental stages (bradyzoite: dark green and tachyzoite: mint) can be seen on top of the genes. Gene identifiers are provided on a per row basis on the right and sample accessions are provided below the heatmap. Rows and columns have been clustered and expression amount is plotted in log2 scale using the pheatmap package in R.

For the expression analysis of *T. gondii* genes and miRNAs, a set of RNA-seq samples was acquired (Croken et al., 2014). Pre-processing of the downloaded RNA-Seq samples produced varying average read lengths (Table 1). This difference in length needed to be taken into account during normalization and nucleotide mapping rather than merely considering read or transcript mapping rates. After the normalization process, the general distribution of gene expression appeared similar among samples despite variation in average read length indicating the effectiveness of the NKMN normalization approach (Supplementary Figure 3). This normalized expression among samples was compared (Supplementary Table 4) and the most expressed 50 genes (top 50 of the average of all 18 samples) are presented in Figure 1. The process of picking the genes most expressed on average identifies the genes that are similarly expressed in all samples. This is confirmed by the heatmap in Figure 1. There is no significant expression difference between developmental stages of *T. gondii* among the most expressed 50 genes. Samples from strains cluster together which reveals that among these genes that are highly expressed in all samples they are more uniformly expressed on a per strain basis than on a per developmental stage basis. Unfortunately, six samples present a slightly different behavior (SRR15429[19,22,25,28,31,34]). Investigation into the origin of this revealed that the outliers have the shortest average read length and the largest percentage of deleted reads after pre-processing (Table 1). However, the overall trend of strain before stage is not influenced by these differences (Figure 1). Furthermore, in both cases cluster analysis shows that PLK and RH have a more similar gene expression among the top 50 genes than CTG.

Figure 1 shows extreme average expression among samples which summarizes the genes that are highly expressed in all of them. This information is useful to detect important genes and perhaps to develop strain/stage independent biomarkers. Differential gene expression in contrast is important to analyze differences among strains and stages.

### Differential gene expression

**Figure 2:**
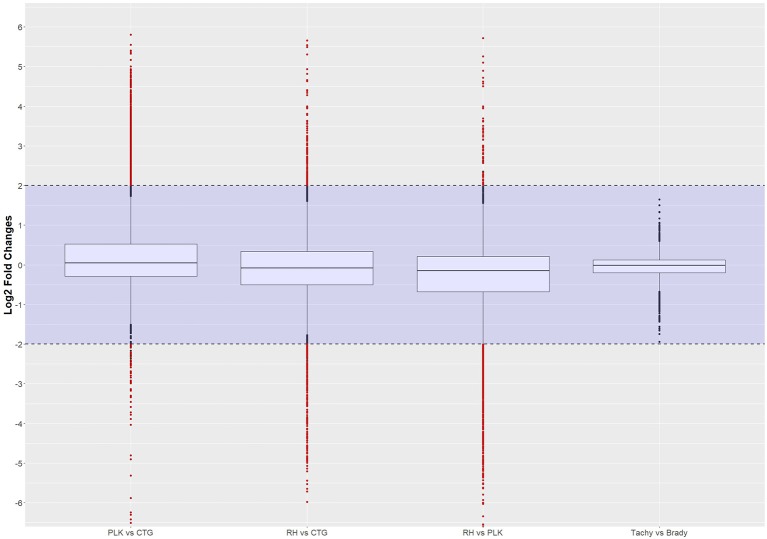
Distribution of differential gene expression (log2 transformed) between strains and developmental stages. Gene expressions falling into the shaded area (violet) were not considered to be differentially regulated. Red dots outside the shaded area also pass the 0.05 *p*-value significance threshold. The number of differentially regulated genes is 529 for PLK vs. CTG, 328 for RH vs. CTG, 613 for RH vs. PLK, and 0 between stages. See Supplementary Table 5 for the list of gene expression.

Differential expression analysis was done in R using the NKMN normalized gene expression and employing *t*-test with Benjamini-Hochberg correction. Only genes expressed in at least 70% of the samples were considered for differential expression analysis. Out of a total of 8,920 annotated genes in *T. gondii*, 7,834 genes (RH), 8,047 genes (PLK), and 7,853 genes (CTG) passed the 70% criteria. For the developmental stages 7,949 genes (tachyzoite) and 7,954 genes (bradyzoite) were available for differential expression analysis after filtering. For the comparison between stages and strains, only these expressed genes in both of them were taken into account, which resulted in a further decrease of comparable genes: 7,790 genes (RH vs. PLK), 7,679 genes (RH vs. CTG), 7,781 genes (PLK vs. CTG), and 7,863 genes (tachyzoite vs. bradyzoite). The log2 transformed distribution of differential expression among strains and stages is displayed in Figure 2.

The distribution of differential expression is most compact and centered on zero for tachyzoites vs. bradyzoites (Figure 2) although the largest number of genes were available for comparison. This is confirming the previous transcriptome analysis of Croken et al. (2014) from which the data for this study was acquired. Distribution of differential expression is similar for RH vs. CTG and PLK while somewhat different for PLK vs. CTG (Figure 2). This further confirms the finding that the type I RH strain and type II PLK strain are closer related in respect to their expressed genes than the type III CTG strain that originates from a cat (Figure 1). Calculated log2 fold changes and adjusted *p*-values were used to further filter the genes for these comparisons. Selected significance threshold for *p*-value was < 0.05 and for the log2 fold change (l2fc), gene expressions with log fold changes <−2 or >2 were applied. While most genes were not differentially expressed given these criteria, 529 genes in the PLK vs. CTG comparison were found to be differentially expressed, whereas differentially expressed genes for RH vs. CTG amounted to 328 and for RH vs. PLK to 613. There was no significantly differentially expressed gene for the comparison between tachyzoite and bradyzoite stages. *P*-values and log fold change values for all comparisons and filtered (by *p*-value and log2 fold change) genes are available in Supplementary Table 5.

**Figure 3:**
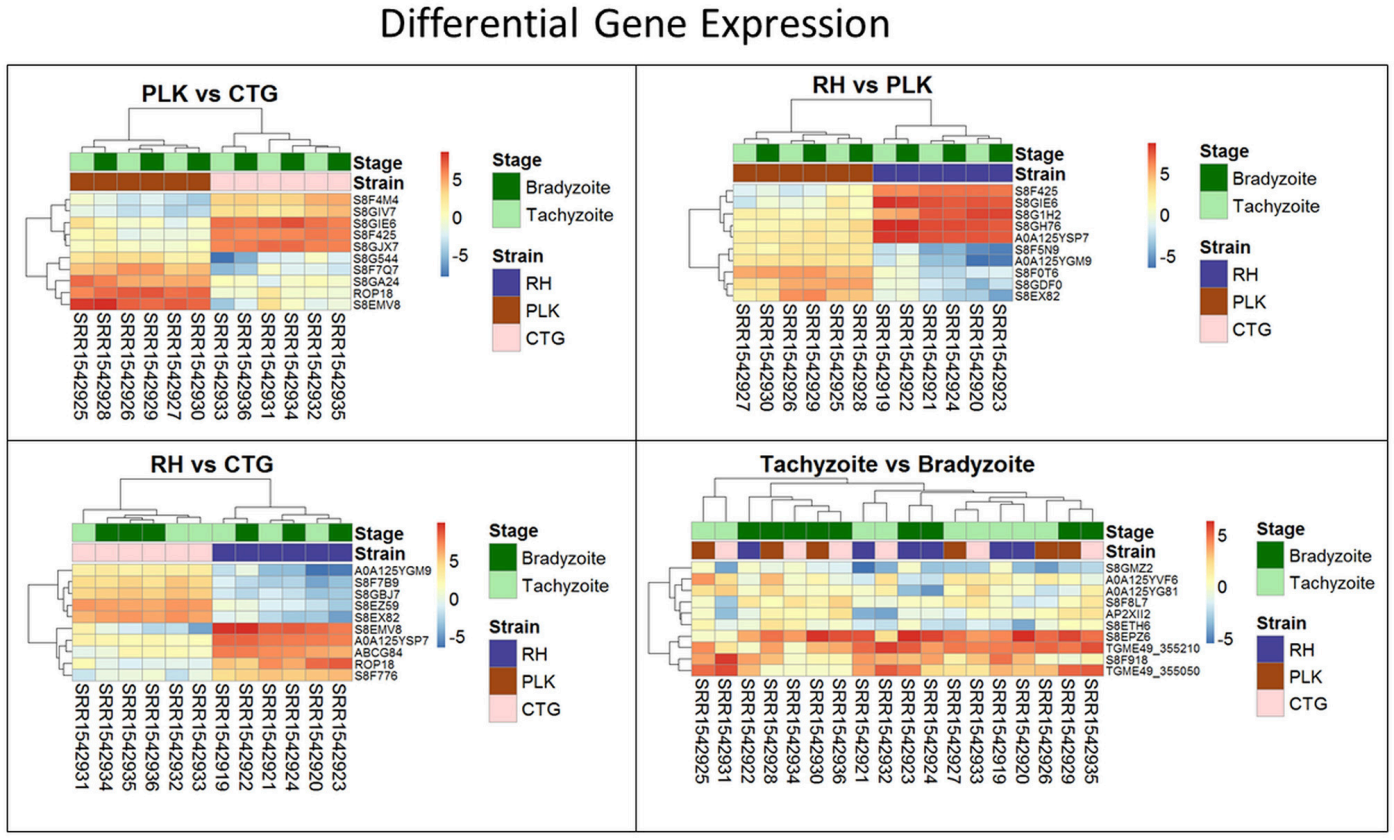
Heatmaps showing the five most overexpressed genes per strain (RH: blue, PLK: brown, CTG: pink) and developmental stage (bradyzoite: dark green, tachyzoite: mint). Each pair presents 10 genes of which 5 are overexpressed in one of the strains/stages and 5 in the other. Genes are hierarchically clustered based on the expression among replicates. Overexpression was analyzed with pooled replicates, but for a better overview, all measurements are shown in columns including their hierarchical clustering. Supplementary Table 5 contains all differential expression results and additional functional annotation.

For each pair of strains, the five most differentially expressed genes per strain were chosen (Figure 3). Only for RH vs. CTG, the differential expression clusters developmental stages while for PLK vs. CTG and RH vs. PLK the stages do not cluster at all. Overexpressed genes in RH are not as strongly overexpressed as for CTG and PLK. For developmental stages, which did not have any significantly differentially expressed genes, the heatmap (Figure 3, bottom right) does not display the expected clustering for strains or developmental stages.

Many of the differently expressed genes are encoding hypothetical proteins making it difficult to draw any conclusion. However, with the ROP18 gene, our analysis showed a significant stronger expression of the rhoptry protein ROP18 in RH and PLK than in CTG confirming previous studies that showed altered ROP18 gene expression in type III strains (Taylor et al., 2006). As has been described, this difference is linked to gene polymorphism and an additional DNA segment upstream of the ROP18 gene in type III strains (Boyle et al., 2008). ROP18 is accepted as an important virulence factor; it acts as a serine-threonine kinase that phosphorylates immunity-related GTPases (IRGs) thereby blocking interferon-gamma-mediated immune responses and preserving integrity of the parasitophorous vacuole membrane especially in mouse-virulent type I and to a lesser extend in type II strains (Fentress et al., 2012; Hermanns et al., 2016; Simpson et al., 2016). Importantly, our finding of strain-specific ROP18 gene expression also serves as proof-of-principle for the *in-silico* analysis of microRNA—mRNA interactions of *T. gondii*.

Another such example is our finding of the NTPase I gene (S8G1H2) being strongly expressed in type I RH strain, but not in the type II PLK strain. In the RH strain, the encoded enzyme exists as a mixture of two isoenzymes termed NTPase I and NTPase II. Whereas, the NTPase II gene is present in all strains of *T. gondii*, the NTPase I gene is restricted to virulent type I strains only. A slower hydrolyzation rate of ADP to AMP for NTPase I in comparison to NTPase II was identified as the functional difference between both isoforms (Asai et al., 1995). This early observation of strain-specific expression of the NTPase I gene has recently also been confirmed by reverse genetic analysis. This technique also allowed to exclude an impact of NTPase I or II on virulence in the mouse model (Olias and Sibley, 2016).

Finally, using our *in-silico* analysis, we identified a calcium-translocating P-type ATPase, PMCA-type protein (S8EZ59) with strain-dependent expression levels. A strain-specific expression of enzymes belonging to these systems has not been described, so far. These systems are involved in signaling by regulation of calcium homeostasis and release (Nagamune et al., 2008). With TgPMA1 and TgPMA2, a P-type ATPase has been identified in *T. gondii* that exists in two isoforms; whereas TgPMA1 is restricted only to bradyzoites, TgPMA2 is expressed also in tachyzoites (Holpert et al., 2001).

### MicroRNA expression

For the expression analysis of miRNAs, pre-miRNAs were compiled into an annotation file to enable usage with standard expression analysis workflows. The same NKMN normalization was applied to miRNA expression analysis. Due to aforementioned differences in average read lengths, raw miRNA counts varied greatly among samples even though normalization was performed and despite the normalization being effective for genes (Supplementary Figure 2). We hypothesize that perhaps mature miRNAs are more likely to be sampled by shorter reads which is further confirmed by the lower mapping ratio of samples with longer reads (Table 1).

**Figure 4:**
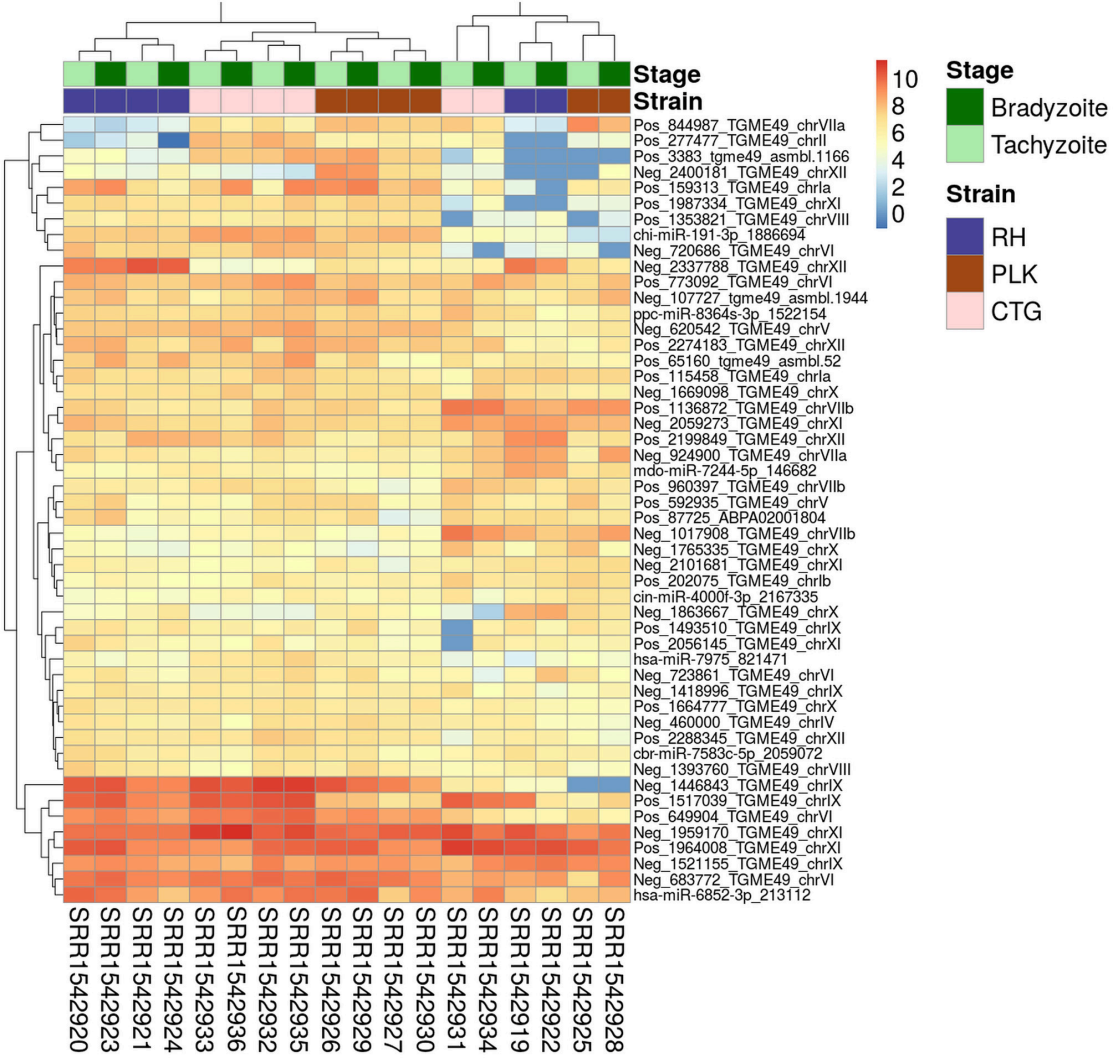
Heatmap showing the 50 miRNAs with largest average expression among samples. The strains (RH: blue, PLK: brown, CTG: pink) and developmental stages (bradyzoite: dark green, and tachyzoite: mint) can be seen on top of the genes. Gene identifiers are provided on a per row basis on the right and sample accessions are provided below the heatmap. Rows and columns have been clustered and expression amount is plotted in log2 scale using the pheatmap package in R.

In a similar fashion to gene expression analysis, the on average most expressed 50 miRNAs were identified for a general idea of expression among miRNAs and samples (Figure 4). A similar picture emerges for miRNAs as for genes with the exception that similarity between strains is not as clear as in the gene expression analysis. CTG and PLK seem closer related in terms of expression for some part of the data and for another part of the data RH and PLK are closer related which is according to expectation (Figure 4). The same samples which were outliers for genes (overall less expression, Figure 1) show the opposite behavior for miRNAs (overall more expression, Figure 4). Also, these samples confirm the closer relationship between RH and PLK seen for genes. Similarly, to gene expression, neither the development stage nor the strain show significant overall differences in expression for the 50 most expressed genes (on average). It is noteworthy, that among the on average most expressed 50 miRNAs, 43 were novel miRNAs predicted in this study whereas only seven of them show high similarity to known miRNAs (Figure 4).

### Differential MicroRNA expression

From initial 1,227,917 predicted miRNA with a model confidence score >0.99, 4,589 miRNAs remained for further analysis. Similar to what was done during gene differential expression analysis, miRNAs were required to be expressed in at least 70% of the samples. For the *T. gondii* strains, this lead to 398 miRNAs (RH), 515 miRNAs (PLK), 401 miRNAs (CTG); and for the developmental stages 448 miRNAs (bradyzoite), and 447 miRNAs (tachyzoite), remained. These numbers further decreased for comparison groups: 272 miRNAs (RH vs. PLK), 258 (RH vs. CTG), 289 (PLK vs. CTG), and 328 (tachyzoite vs. bradyzoite).

**Figure 5:**
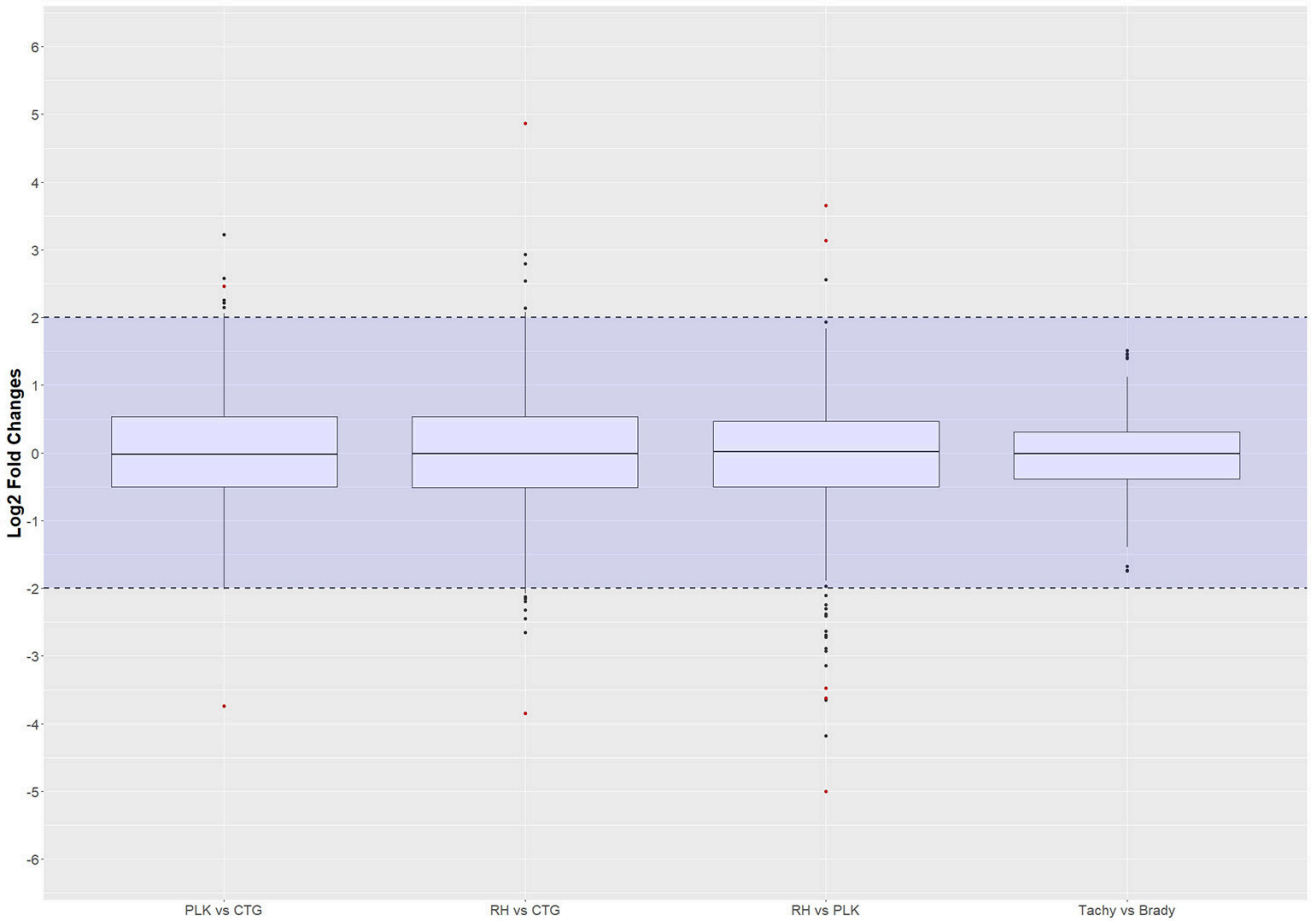
Distribution of differential miRNA expression (log2 transformed) between strains and developmental stages. MicroRNA expressions falling into the shaded area (violet) were not considered to be differentially regulated. Red dots outside the shaded area also pass the 0.05 *p*-value significance threshold. The number of differentially regulated miRNAs is 2 for PLK vs. CTG, 2 for RH vs. CTG, 5 for RH vs. PLK, and 0 between stages. See Supplementary Table 6 for the list of miRNA expression.

For these miRNAs, Benjamini-Hochberg corrected *t*-test was applied and log2 fold changes were calculated using R. The same threshold values as for the differential gene expression analysis (*p*-value < 0.05, l2fc > 2 or l2fc < −2) were applied to differential miRNA expression analysis. Only 2 miRNAs were found to be differentially expressed between PLK and CTG, another 2 miRNAs between RH and CTG and 5 in RH vs. PLK. No significantly differentially expressed miRNAs were found for tachyzoite vs. bradyzoite stages which is in-line with the findings for the above gene expression analysis. The distribution of log2 fold changes for miRNAs can be seen in Figure 5, and the list of miRNAs with their *p*-values and log2 fold changes is available in Supplementary Table 6.

The log2 fold change distributions among strains and stages is quite similar for miRNAs (Figure 5) with the exception of differential expression for tachyzoites vs. bradyzoites which shows a very small inter quartile range (Figure 5).

**Figure 6:**
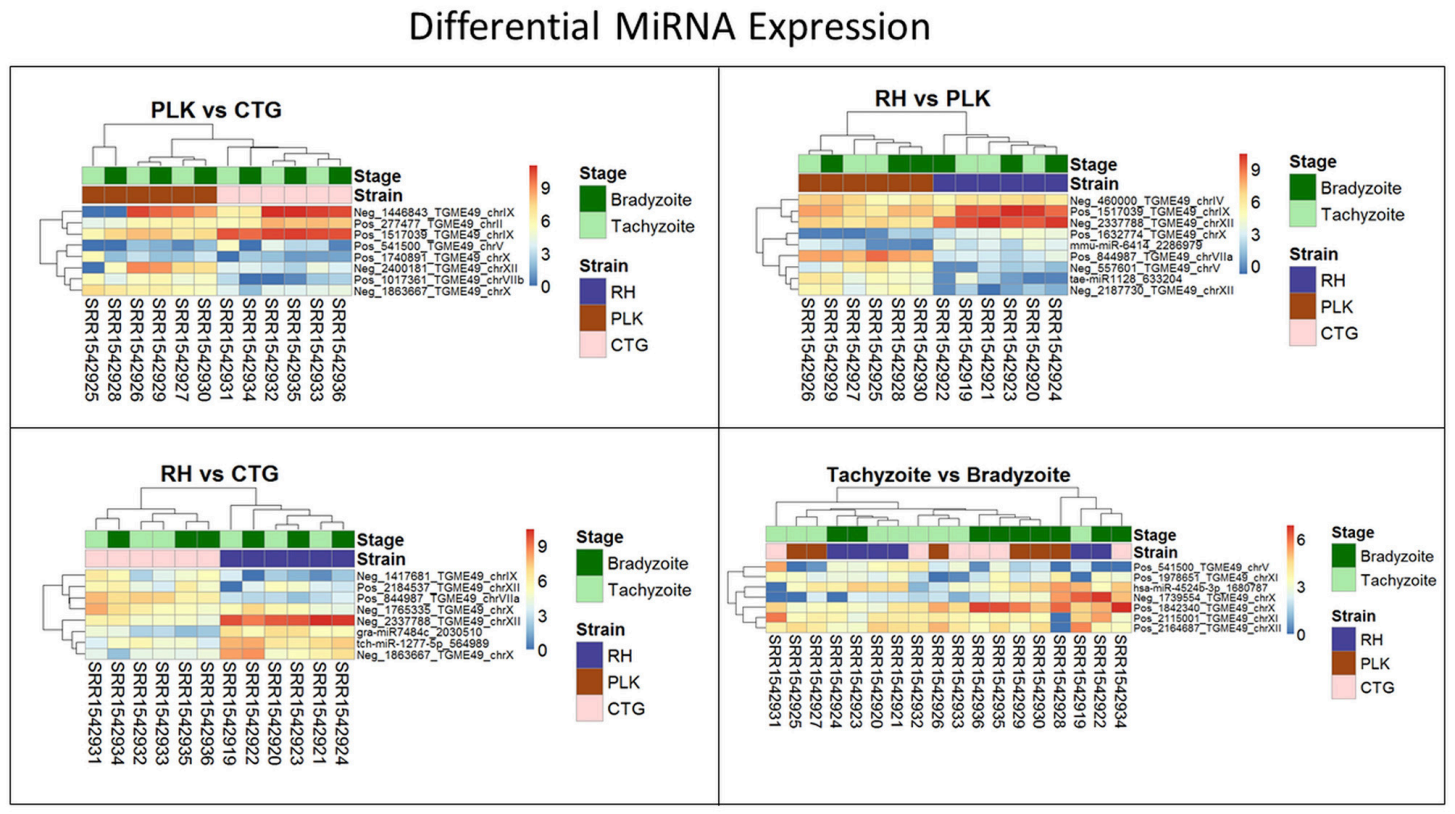
Heatmaps showing all differentially expressed microRNAs for pairs of strains (RH: blue, PLK: brown, CTG: pink) and developmental stages (bradyzoite: dark green, tachyzoite: mint). MicroRNA expression is hierarchically clustered based on the expression among replicates. Overexpression was analyzed with pooled replicates, but for a better overview, all measurements are shown in columns including their hierarchical clustering. Supplementary Table 6 contains all differential expression results and additional functional annotation.

Clustering of strains is dominating clustering in respect to developmental stage (Figure 6). Four of the miRNAs were annotated via similar sequences in miRBase, but unfortunately, they are either of plant origin or their targets are not annotated so that a cross annotation is not possible in this case. Many of the significantly differentially expressed miRNAs were detected in this study. While differential miRNA and gene expression are topics for complete manuscripts, we believe that instead miRNA—mRNA interactions which define the regulatory process are more worthy of discussion.

### MicroRNA—mRNA interactions

For miRNAs to be functionally active, they need to be co-expressed with their target mRNAs. It is, therefore, prerogative to ensure that both miRNA and target(s) are expressed in the same sample to enable any conclusion about miRNA regulation. Above, pre-miRNAs and mature miRNAs were detected and their expression was confirmed. Gene expression was also established for the same samples. Therefore, it is possible to analyze miRNA and mRNA co-expression in this study. As a caveat, even though miRNA expression was explored in this study, generally miRNA expression analyses require specifically prepared libraries (Eminaga et al., 2013). However, the samples used in this study were prepared to detect mRNAs rather than miRNAs which led to low detection of miRNAs and almost no detection of their differential expression. To overcome this challenge, all miRNAs that do not originate from an annotated gene were discarded. For the remaining miRNAs (4,240) the expression of their source genes was used to represent their expression. Naturally, more reads will be mappable to mRNAs than to much shorter miRNAs which makes the approach chosen here more robust, as well. Thus, an interaction for this study is defined by a source gene and a target gene connected by via miRNA and co-expressed in the same sample.

**Figure 7:**
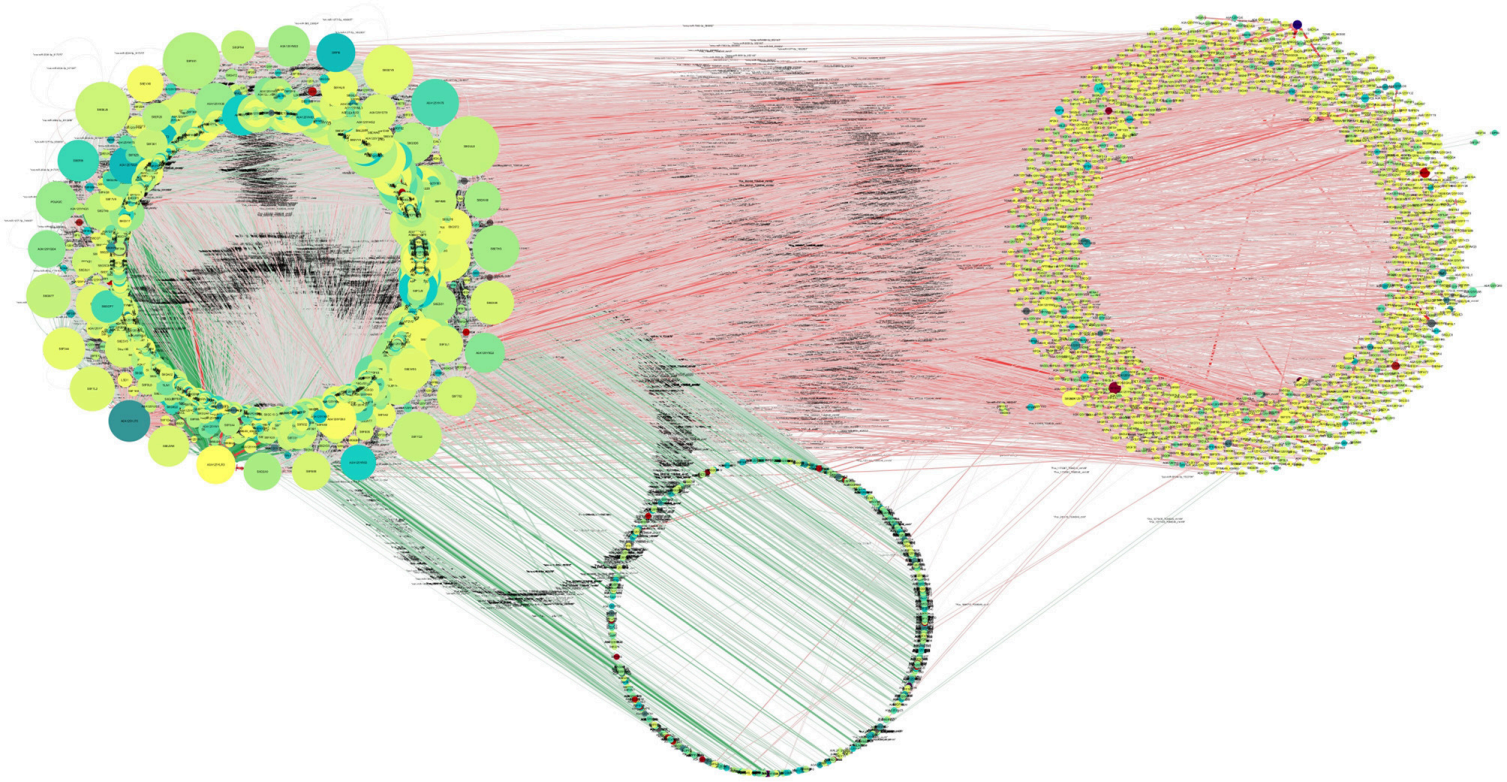
Putative functional interactions. All of the co-expressed interactions found in this study are shown as a network. This network will be further analyzed in future work to draw better and specific conclusions about *T. gondii* miRNA-based regulation. Further information can be found in Supplementary Table 8, where all targets of miRNAs are listed.

Overall, 4,240 miRNAs and 8,920 (all annotated *T. gondii* mRNAs) were available for interaction analysis. Theoretically, ~40 million such interactions are possible given these data. The first restriction applied to the data was that any interaction considered here needed to have the miRNA and its target co-expressed in at least one sample. Out of a total of 161,970 interactions 65,602 were found to be co-expressed in this manner (Figure 7).

For the analysis of the differential expression of interactions, the ratio of target gene expression divided by source gene expression was used. Interactions needed to exist in at least 70% of the samples in order to qualify for differential expression analysis.

Out of the total 65,602 interactions found, 63,120 of them were expressed in the samples of the RH strain. PLK had 63,778, CTG strain 62,494, bradyzoite stage 62,994, and tachyzoite stage 62,369 interactions in their respective samples. As before, when considering differential expression of interactions, these numbers further trimmed down to 62,867 (RH vs PLK), 61,923 (RH vs. CTG), 62,441 (PLK vs. CTG), and 61,874 (tachyzoite vs. bradyzoite).

**Figure 8:**
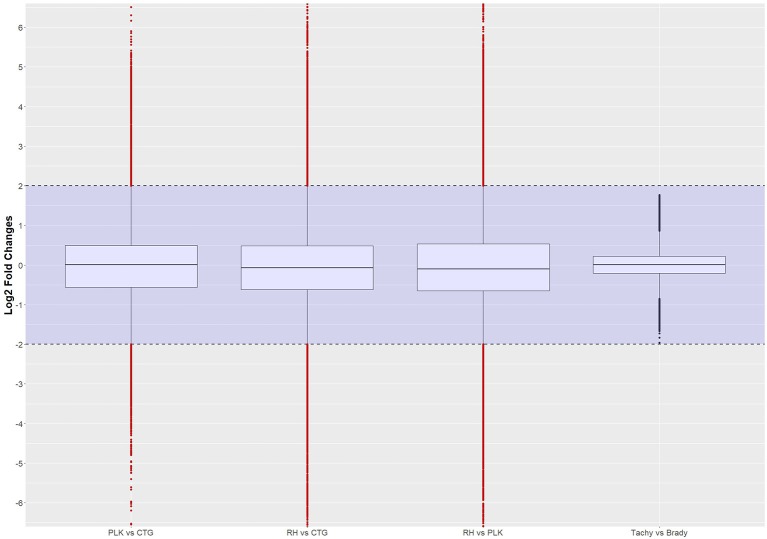
Distribution of differential expression of interactions (log2 transformed) between strains and developmental stages. Expressed interactions falling into the shaded area (violet) were not considered to be differentially regulated. Red dots outside the shaded area also pass the 0.05 *p*-value significance threshold. The number of differentially regulated interactions is 4502 for PLK vs. CTG, 5488 for RH vs. CTG, 6508 for RH vs. PLK, and none between stages. See Supplementary Table 7 for the list of expressed interactions.

*T*-test and log2 fold change calculations were performed for the remaining interactions. Thresholds were kept the same (*p* < 0.05, l2fc < −2 or l2fc > 2) for the assessment of differential expression among strains and development stages. As can be seen in Figure 8, log2 fold changes between developmental stages did not vary significantly which is similar to the findings for differential expression of genes and miRNAs. The distributions look similar to the distributions of differential gene expression (Figure 2) as can be expected since an interaction is defined by the ratio of the expression of a pair of genes. After significance filtering, 4,502 interactions were found to be differentially expressed in PLK vs. CTG, 5,488 (RH vs. CTG), 6,508 (RH vs. PLK), and none for bradyzoites vs. tachyzoites. The list of these interactions, log2 fold change, and *p*-values is available in Supplementary Table 7.

**Figure 9:**
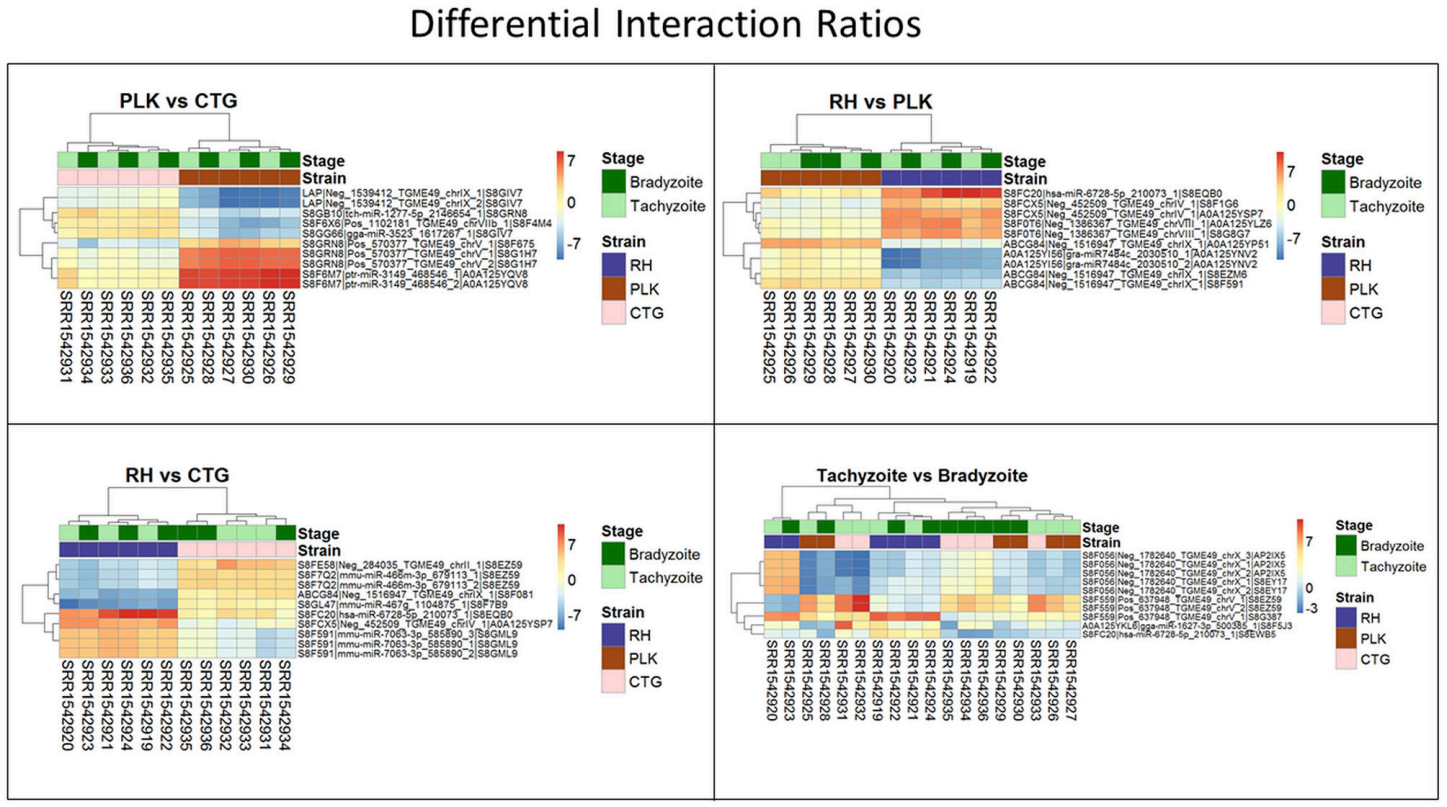
Heatmaps showing the five most overexpressed interactions per strain (RH: blue, PLK: brown, CTG: pink) and developmental stage (bradyzoite: dark green, tachyzoite: mint). Each pair presents 10 interactions of which five are overexpressed in one of the strains/stages and 5 in the other. Interactions are hierarchically clustered based on the expression among replicates. Overexpression was analyzed with pooled replicates, but for a better overview, all measurements are shown in columns including their hierarchical clustering. Supplementary Table 7 contains all differential expression results and additional functional annotation.

Most of the top differentially expressed interactions are new detections in this study. For the comparison between RH and CTG, however, more than half of the top differentially expressed interactions involve miRNAs similar to mouse examples. The comparison among strains leads to clustering of strains before stages (Figure 9). This is different for comparison between developmental stages where the clustering is not as expected which may be due to missing of actual significant differential expression. The complete list of interactions, available in Supplementary Table 7, may path the way for new findings about regulation within *T. gondii*.

It is interesting to note, that 10s of miRNAs, 100s of genes, and 1000s of interactions were significantly differentially expressed among strains but not between developmental stages. The interactions consist of co-expressed miRNAs and genes. From these results it can be deduced that few miRNAs and genes can lead to a wide variety of expressed (including differentially expressed) interactions which may have a large influence on the resulting phenotype. To the best of our knowledge, this is the first study investigating expressed interactions.

Throughout this analysis it was found that tachyzoites and bradyzoites were extremely similar. This is in contrast to previous findings. For example, LDH1 has been described to be expressed only in tachyzoites and LDH2 only in bradyzoites (e.g., Abdelbaset et al., 2017) while our results imply that LDH1 is uniformly expressed and LDH2 only slightly and not significantly differentially expressed between developmental stages (Table 2). No significant evidence for GRA5 dysregulation could be found although it has been reported to be upregulated in RH bradyzoites and downregulated in PLK bradyzoites (Cleary et al., 2002).

**Table 2:** Differential expression values for selected genes.

	**Differential expressions**					
**Gene name**	**LDH1**		**LDH2**		**GRA5**	
	**L2fc**	***p*** **adjusted**	**L2fc**	***p*** **adjusted**	**L2fc**	***p*** **adjusted**
PLK vs. CTG	−0.919	0.0455	0.254	0.8707	−0.860	0.2811
RH vs. CTG	−0.225	0.4964	−4.576	0.0056	0.429	0.2957
RH vs. PLK	0.694	0.1023	−4.830	0.0030	1.289	0.1026
Tachy vs. Brady	−0.034	0.9135	−1.380	0.9919	0.140	0.9474
**Gene name**	**ROP18**		**S8G1H2**		**S8EZ59**	
	**L2fc**	***p*** **adjusted**	**L2fc**	***p*** **adjusted**	**L2fc**	***p*** **adjusted**
PLK vs. CTG	6.862	0.0003	−0.597	0.5698	−1.431	0.0677
RH vs. CTG	6.758	0.0005	4.664	0.0030	−7.431	0.0009
RH vs. PLK	−0.103	0.9251	5.260	0.0010	−6.000	0.0004
Tachy vs. Brady	0.317	0.9135	0.362	0.9830	0.436	0.9849

*Listed genes in this table were found to be differentially expressed in other studies. While this was not the case in our study for all of them, it was found that some of the genes still show such differences (LDH2, ROP18, and S8G1H2)*.

Our results do confirm a slight downregulation of GRA5 in bradyzoites, albeit, without significance (Table 2). The data used in this study derived from Croken et al. (2014) and they also identified no significant expression differences between developmental stages. This is in agreement with a recent study performing principle component analysis which led to a close grouping of tachyzoites and bradyzoites stages (Behnke et al., 2014). Further works, which must also proof the proper induction of bradyzoites, are necessary to be able to better understand differential gene, miRNA, and miRNA interaction expression between developmental stages.

## Conclusion

Little is known about the miRNA-based regulation in *T. gondii*. Therefore, pre-miRNAs, their associated mature miRNAs, and their mRNA targets were predicted from the ME49 genome. A publicly available RNA-seq dataset investigating three *T. gondii* strains (RH, PLK, and CTG) and two developmental stages (tachyzoite and bradyzoite) was used to analyze expression of the detected miRNAs and their targets. In an attempt to add further confidence, miRNAs and their targets were analyzed together in form of putative interactions. 65,602 expressed interactions were found between the 4,240 miRNAs and 8,920 annotated mRNAs. Previously, 339 miRNAs have been described for *T. gondii* of which we previously disputed 47 out of 339 (Saçar Demirci et al., 2016). Here we present 4,240 miRNAs (containing 305 of the 339 known ones) and their targets co-expressed in the same sample (Supplementary Table 8). It is our contention, that interactions have a higher confidence than considering merely miRNAs. These interactions are likely to represent interesting drug targets. For instance, miRNA mimics (Wang, 2011) can be used to target the targeting-side of interactions while miRNA decoys (Bak et al., 2013) can be designed to target the miRNAs themselves. The former introduces miRNAs which reduce the amount of available protein of their targets and they may be mimics of existing *T. gondii* miRNAs, but can also be designed *de novo*. The latter reduces the impact of intrinsic miRNAs by providing an abundance of target sequences effectively reducing the amount of interactions with mRNAs. Such approaches have been tested for example in human (Avci and Baran, 2014) and since *T. gondii* exchanges vesicles with its hosts (Romano and Coppens, 2013), drugs may be delivered to the parasite via the host system. Knowledge about the interactions in different stages of *T. gondii* development and within different hosts will enable the design of miRNA mimics and decoys by showing their availability and differential distribution.

## Author contributions

JA had the initial idea which was developed in a project together with UG. MS and İA performed the computational studies and together with JA and UG interpreted the results. UG put the results in their biological context. The manuscript was written with contributions of all authors, and all authors read and approved the final version.

### Conflict of interest statement

The authors declare that the research was conducted in the absence of any commercial or financial relationships that could be construed as a potential conflict of interest.

## Supplementary material

Supplementary Table 1 Statistics of reads. All of the mapped, unmapped, and deleted reads can be seen in their respective columns.

Supplementary Table 2 List of pre-miRNAs. From 0.99 confidence to all filtering steps, lists of pre-miRNAs are provided in their respective sheets.

Supplementary Table 3 List of all mature miRNAs. Mature miRNAs that passed the filtering after machine learning process are given with their sequences.

Supplementary Table 4 Raw and normalized gene and miRNA counts. All of the count values are presented in their respective samples in columns, in 4 different sheets for raw and normalized values.

Supplementary Table 5 List of differentially expressed genes. All of the comparisons are given in different excel sheets and the significant differential expressions are gathered in extra sheets for each comparison.

Supplementary Table 6 List of differentially expressed miRNAs. All of the comparisons are given in different excel sheets and the significant differential expressions are gathered in extra sheets for each comparison.

Supplementary Table 7 List of differentially expressed interactions. All of the comparisons are given in different excel sheets and the significant differential expressions are gathered in extra sheets for each comparison.

Supplementary Table 8 List of mature sequences and their target candidates. For the formed network, all of the targeted genes for each mature miRNA (that survived the filtering) were presented.

Supplementary Figure 1 Score distributions of 1,000 machine learned models established using 1,000-fold Monte Carlo cross validation.

Supplementary Figure 2 Distribution of normalized miRNA expressions. Normalization method that was employed to genes were applied to miRNA expressions. It was seen that median values were varying between samples but closer among similar mean read lengths.

Supplementary Figure 3 Distribution of normalized genes. The values shown on the y-axis is the resulting numbers from the formula presented in normalization method. Normalization was done for each gene in each sample and the distribution of mapped nucleotides were found to have closer median values than raw counts.
