# Supplementary material for: The Expressed MicroRNA—mRNA Interactions of Toxoplasma gondii
Source: Front Microbiol. 2018 Jan 4;8:2630. doi: 10.3389/fmicb.2017.02630 (PMC5759179; doi:10.3389/fmicb.2017.02630)
Supplement: Supplementary Figure 1 — Score distributions of 1,000 machine learned models established using 1,000-fold Monte Carlo cross validation. [file DataSheet1.DOCX]

MicroRNA Regulation in Toxoplasma gondii

**İlhan Erkin Acar, Müşerref Duygu Saçar Demirci, Uwe Groß, Jens Allmer**

Biotechnology, Izmir Institute of Technology, Urla, Izmir, Turkey

Molecular Biology and Genetics, Izmir Institute of Technology, Urla, Izmir, Turkey

Medizinische Mikrobiologie, Universitätsmedizin Göttingen, Göttingen, Germany

# Abstract

MicroRNAs (miRNAs) are involved in post-transcriptional modulation of gene expression and thereby have a large influence on the resulting phenotype. We have previously shown that miRNAs may be involved in the communication between *Toxoplasma gondii* and its hosts and further confirmed a number of proposed specific miRNAS. Yet, little is known about the internal regulation of miRNAs in *T. gondii*. Therefore, we predicted pre-miRNAs directly from the ME49 genome and filtered them. For the confident hairpins, we predicted the location of the mature miRNAs and established their target genes. To add further confidence, we evaluated whether the hairpins and their targets were co-expressed. Such co-expressed miRNA and target pairs define a functional interaction. We extracted all such functional interactions and analyzed their differential expression among strains (CTG, PLK, and RH) and between two stages (Tachyzoites and Bradyzoites). Overall, we found NNN interactions of which NNN are differentially expressed among strains and NNN are differentially expressed between developmental stages. Since miRNAs and target decoys can be used as therapeutics we believe that the list of interactions we provide will lead to novel approaches in the treatment of toxoplasmosis.


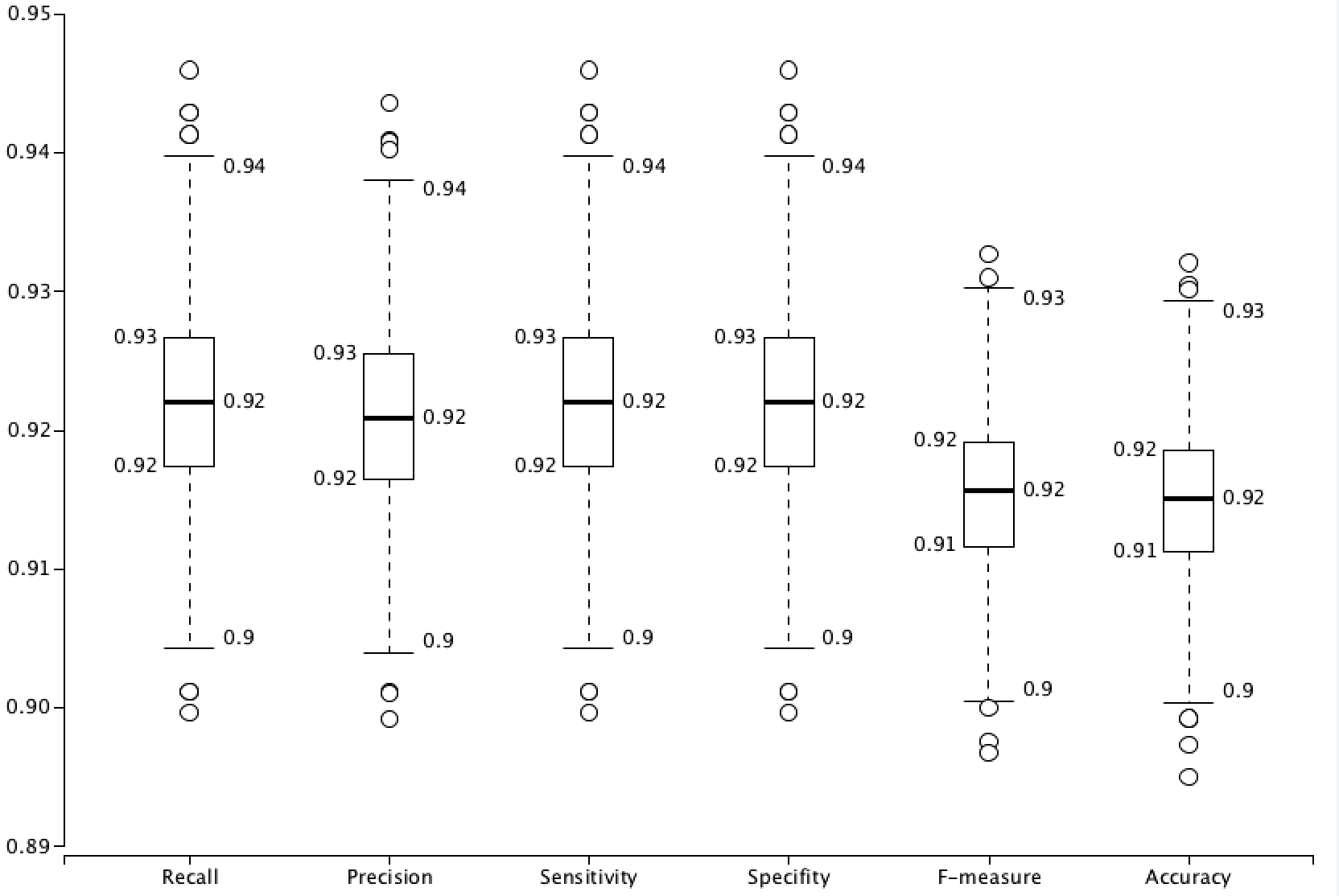


Figure 1: Score distributions of 1000 machine learned models established using 1000 fold Monte Carlo cross validation.


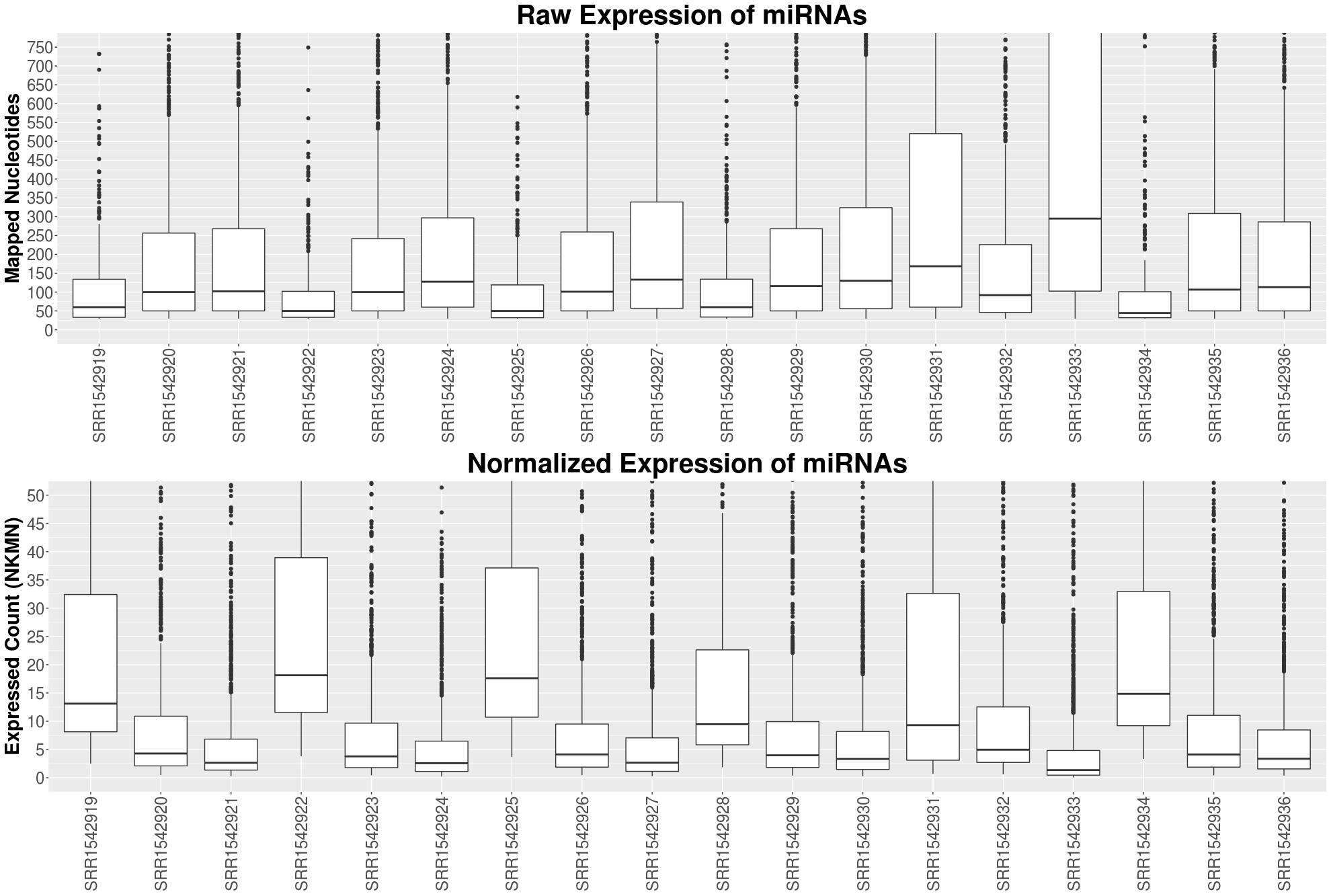


Figure 2. Distribution of normalized miRNA expressions. Normalization method that was employed to genes were applied to miRNA expressions. It was seen that median values were varying between samples but closer among similar mean read lengths.


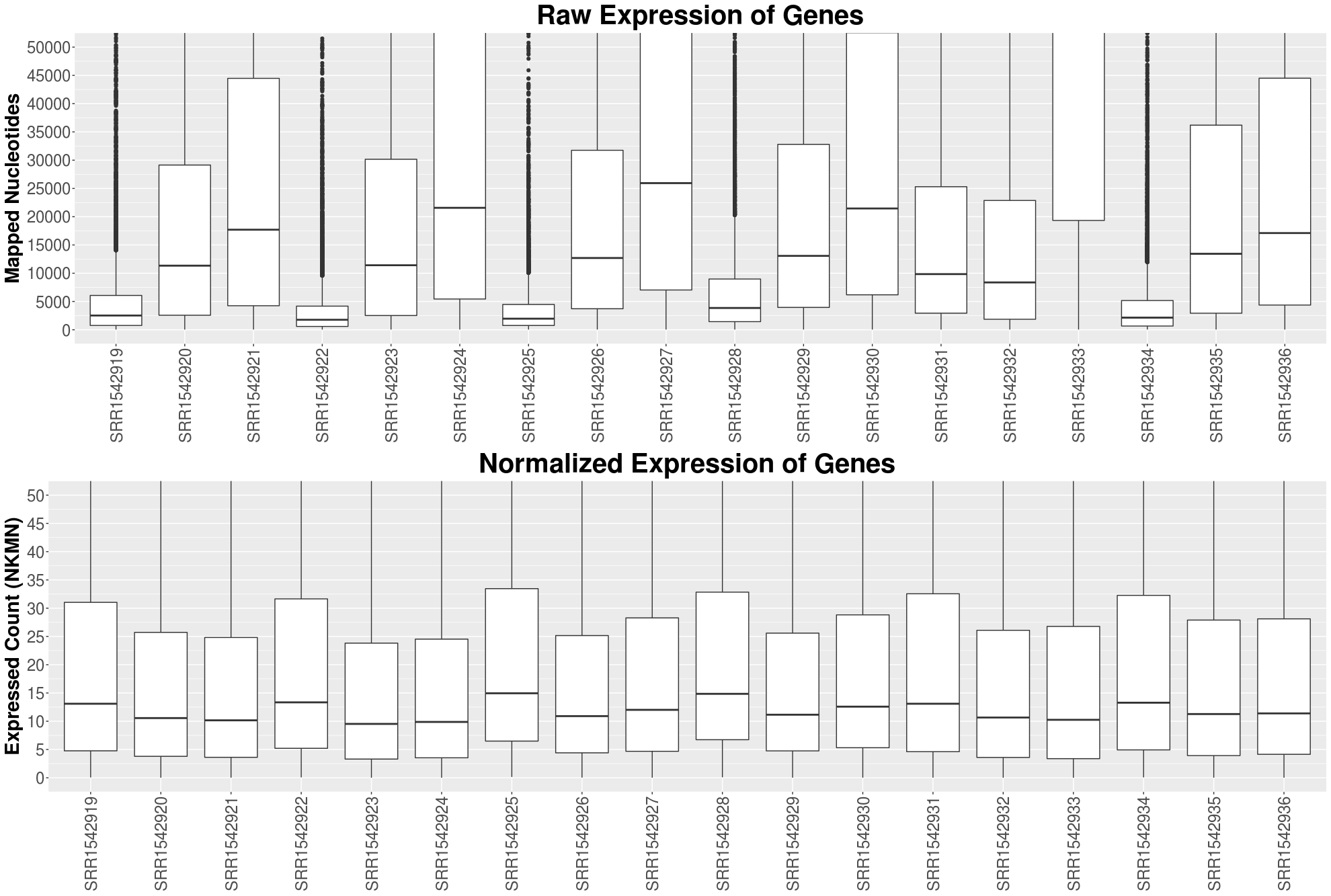


Figure 3. Distribution of normalized genes. The values shown on the y-axis is the resulting numbers from the formula presented in normalization method. Normalization was done for each gene in each sample and the distribution of mapped nucleotides were found to have closer median values than raw counts.
